# In vitro assessment of the antifungal effects of neem powder added to polymethyl methacrylate denture base material

**DOI:** 10.4317/jced.55458

**Published:** 2019-02-01

**Authors:** Shorouq-Khalid Hamid, AlAnoud-Hamad Al-Dubayan, Heba Al-Awami, Soban-Qadir Khan, Mohammed-Moustafa Gad

**Affiliations:** 1 Undergraduate student, College of Dentistry, Imam Abdulrahman Bin Faisal University, P.O. Box 1982, Dammam 31411, Saudi Arabia; 2 MSC, Lecturer of Biostatistics, Department of Clinical Affairs, College of Dentistry, Imam Abdulrahman Bin Faisal University, P.O. Box 1982, Dammam 31411, Saudi Arabia; 3 BDS, MSc, Lecturer, Department of Substitutive Dental Sciences, College of Dentistry, Imam Abdulrahman Bin Faisal University, P.O. Box 1982, Dammam 31411, Saudi Arabia

## Abstract

**Background:**

Denture with antimicrobial activities is desirable to prevent *Candida albican* adhesion subsequently decreasing the susceptibility of denture stomatitis incidence. *Azadirachta Indica*, commonly known as Neem powder has antimicrobial effect but the effect of its addition to acrylic denture base on *C. albicans* adhesion has not been investigated. The aim of this study was determine whether adding neem powder to acrylic denture base materials could reduce *Candida albicans*adhesion.

**Material and Methods:**

One hundred and twenty acrylic resin denture specimens were fabricated and divided into heat-polymerized (n=60) and auto-polymerized (n=60) groups. Each group was further divided into 6 groups (n=10) based on the neem concentration: 0, 0.5, 1, 1.5, 2 and 2.5 wt% of the polymer. After polymerization, the specimens were polished, stored in distilled water, sonicated, sterilized, submerged in artificial saliva containing C. albicans, and finally, placed in an incubator at 37°C. Slide counting and direct culture methods were used to assess the antifungal effects of the neem addition. An analysis of variance and post hoc Tukey’s test were performed for the data analysis (*p*≤0.05 was statistically significant).

**Results:**

Based on the results, the neem addition significantly decreased the *C. albicans* count when compared to the control group (*p*≤0.05). Moreover, the count decreased as the neem concentration increased (lowest count with 2.5 wt%).

**Conclusions:**

The results suggest that adding neem powder to acrylic resin denture base materials reduces the adhesion of *C. albicans*; therefore, the incorporation of neem could be a possible denture stomatitis prevention method.

** Key words:**Denture stomatitis, Candida albicans, Azadirachta indica, neem powder, denture base.

## Introduction

Denture stomatitis (DS) is a widespread disease occurring in both partial and complete denture wearers that tends to affect the mucosa of the palate (1). Poor oral and denture hygiene, a low saliva flow, oral mucous membrane trauma and microbial infections (mostly Candida albicans) are all associated with the development of DS (2,3). Previous investigations have been conducted to determine the role of *C. albicans* in DS, and the results have suggested that it is the main fungal source and a key factor in the pathogenesis of DS. Moreover, it has the ability to develop into a biofilm (4,5). The denture base material itself has poor surface properties that allow *C. albicans* to adhere to and colonize the surface and develop into a biofilm (4,6). 

 In approximately 30–75% of denture wearers, DS has been recognized with a high rate of recurrence, even with antifungal therapy (7). Therefore, different management methods have been used to inhibit fungal growth on the denture base materials, such as the incorporation of antimicrobial medications into the denture lining (8) or tissue conditioning materials (9), and the inclusion of antimicrobial agents in the denture base resin materials (10). Recently, the inhibitory effects on *C. albicans* growth have been investigated experimentally using natural products, such as henna and thymoquinone (TQ), and mixing them with denture base materials (11-13). Nawasrah *et al.* (11), reported a significant reduction in the *C. albicans* count by incorporating a 1% concentration of henna powder into the denture material. Moreover, the antifungal effects of TQ incorporated into polymethyl methacrylate (PMMA) denture base materials were investigated by Al-Thobity *et al.* They concluded that the addition of TQ could be effective for preventing *C. albicans* adhesion and proliferation on the denture surface as one possible method for preventing DS (13).

Among the natural products that have antimicrobial effects is neem, which comes from the *Azadirachta indica* tree (14-16). Neem trees are native to the Indian subcontinent, and various parts and products derived from them are used in traditional medicine (14,15,17). Worldwide interest in A. indica has developed due to the active compounds that it produces, which have been researched over the past sixty years in order to determine the biological activities that are responsible for its medicinal effects (15). It has been reported that A. indica products exhibit immunity enhancing, antidiabetic, anti-inflammatory, antifungal, antimalarial, antibacterial, antiviral and anticarcinogenic properties (14-18). In one recent study, Barua et a., reported that A. indica leaves play a role in fungal growth prevention when incorporated into a tissue conditioner material (19).

Although neem does have antifungal effects, as proven by many studies conducted in the medical and dental fields, its antifungal activity in denture base materials has not yet been investigated. Therefore, the aim of the present study was to evaluate the effects of incorporating neem into PMMA denture base materials on *C. albicans* as a new approach for DS prevention and treatment. The null hypothesis of this study was that the incorporation of neem powder into PMMA denture base materials would not decrease *C. albicans* adhesion.

## Material and Methods

The current study was conducted after obtaining ethical approval by the Ethics Committee for Dental Research, Faculty of Dentistry, Imam Abdulrahman Bin Faisal University.

-Specimen Preparations

Fresh neem leaves were collected locally and air-dried in the shade. Neem powder was prepared from 250 g of the naturally dried leaves using a microgrinding machine. In order to collect only the finest neem powder and remove the coarse particles, the ground up powder was filtered using microfiltration paper. An electronic balance was used to weigh the neem powder so that it could be added to the acrylic powder at concentrations of 0.5, 1, 1.5, 2 and 2.5 wt%. The acrylic/neem powder was gently mixed by hand using a mortar and pestle. Then, the mixture was stirred with an electric mixer at a rotating speed of 400 rpm at room temperature for 30 min in order to obtain a homogenous distribution of the neem powder.

Sixty heat-polymerized acrylic resin specimens were prepared to simulate the denture base fabrication steps. The samples were prepared using negative metal molds with specific dimensions (10×10×3±0.1 mm). They were covered with modeling wax (Vertex Dental B. V., Soesterberg, Netherlands) and invested in type III dental stone (Fujirock EP; GC Corporation, Tokyo, Japan) within a 61B Two Flask Compress (Handler Manufacturing, Westfield, NJ, USA). Next, the wax was melted away to create mold spaces using a wax elimination machine, and a separating medium (162-800-00; Vandex Isoliermittel GmbH, Hamburg, Germany) was applied to all of the stone surfaces while the stone was still warm. The prepared heat-polymerized acrylic resin (BMS 014 powder; BMS Dental, Capannoli, PI, Italy) was mixed according to the manufacturer’s instructions. Then, it was packed (in the dough stage) into the previously created mold spaces under pressure. It was kept in the flask clamps for 1 h before processing. The flasks were processed using the conventional curing method by placing them into a thermal curing unit (KaVo Elektrotechnisches Werk GmbH, Biberach, Germany) for 8 h at 74°C, and then, increasing the temperature to 100°C for 1 h.

The auto-polymerized acrylic resin (BMS 015 powder; BMS Dental) was prepared with the neem concentrations the same way that the heat-polymerized acrylic powder was prepared ( Table 1). A total of 60 standard neem modified auto-polymerized acrylic resin specimens were fabricated in the multiple neem powder concentrations. According to the manufacturer’s recommendations, the polymer/monomer was mixed and packed directly into the molds. When the glaze of the repair material surface was lost, the molds and their contents were placed into a pressure chamber with water at 40°C and a pressure of 30 lb/in2 for 15 min.

**Table 1:**
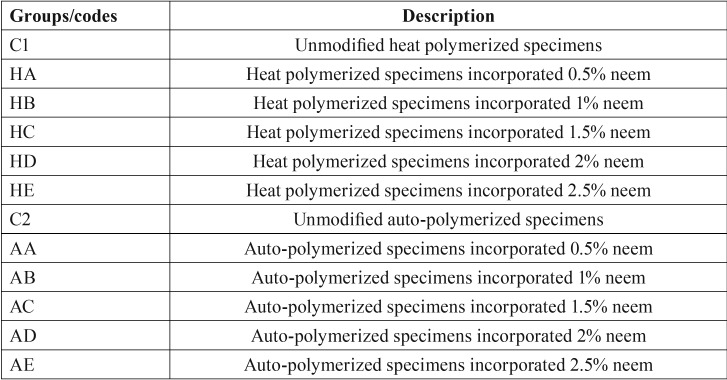
Tested groups and description according to neem concentrations.

After the complete polymerization of both materials, the specimens were finished and polished using a tungsten carbide bur (HM 79GX-040 HP; Meisinger USA, Centennial, CO, USA) with a thin cross-cut at 18,000 rpm, followed by a coarse grain cylindrical rubber top bur (specifically for acrylic resin) (Super Acrylic Polisher; Long Dental), and then, a fine grain cylindrical rubber top bur (Super Acrylic Polisher; Long Dental) (20). In order to standardize the samples, a mechanical polisher (MetaServ 250 grinder-polisher; Buehler GmbH, Braunschweig, Germany) was used for the final polishing with a polishing cloth disc (TexMet C10 in, 42-3210; Buehler GmbH) and polishing suspension (0.05 microns) (MasterPrep polishing suspension; Buehler GmbH) at a speed of 100 rpm for 2 min in wet conditions. A digital caliper (with 0.01-mm accuracy) (Neiko 01407A Electronic Digital Caliper) was used to check the dimensions of the specimens; then, they were cleaned with soap and water using a regular toothbrush, followed by a steam jet. Finally, all of the specimens were stored in distilled water at 37oC for 48 h, after which, they were subjected to the microbiological testing.

-*C. albicans* adherence assay

Prior to the Candida adhesion experiment, the specimens were cleaned in an ultrasonic cleaner using water and a detergent bath for 15 min. Then, they were sonicated in distilled water for 15 m and air-dried. All of the specimens were immersed in artificial saliva containing 2,000,000 *C. albicans* cells (ATCC 10231) ( Table 2) for two d at a temperature of 37oC.

**Table 2:**
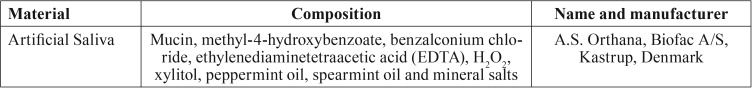
Composition of artificial saliva.

The nonadherent *C. albicans* cells were removed from the acrylic plates by washing them three times with phosphate-buffered saline (PBS). Then, they were placed in sterile tubes with 1 ml of Sabouraud dextrose broth (Acu Medica Lab Systems Ltd., Mumbai, Maharashtra, India) for 48 h. The adherent *C. albicans* cells were dislodged from the acrylic specimen surfaces by scraping them. They were vortexed for 10 min, followed by centrifugation at 4,500 rpm for 5 min. Then, the effectiveness of each disinfecting agent concentration was evaluated using 2 different methods.

Direct Culture Method (Colony Forming Units)

First, a 100 µl sample was obtained from each centrifuge tube. The sample was serially diluted, spread onto a petri dish, and incubated for 48 h at 37°C. After incubation, the number of *C. albicans* colonies in each quadrant in which acceptable growth was noted was counted using a colony counter pen (SP Scienceware, Bel-Art Products Inc., Pequannock, NJ, USA), with the final number corrected for the dilution factor.

-Slide Counting Method

A volume of 7.5 μl was acquired from each concentrated pellet and added to 2.5 μl of a trypan blue solution in 0.4% PBS (MP Biomedicals, LLC, Santa Ana, CA, USA). Then, a microscopic evaluation of the new 10 μl solution was conducted after placing it on a slide counter (Neubauer Slide Counter; Paul Marienfeld GmbH & Co. KG, Lauda-Königshofen, Germany). The trypan blue stain can distinguish between living and dead *C. albicans*. The living *C. albicans* is transparent with a blue peripheral line, while the dead *C. albicans* usually appears blue. A slide counter contains 4 major squares that are each divided into 16 smaller squares. The *C. albicans* was counted on 2 major squares and multiplied by 2 to obtain the total *C. albicans* count on each slide, which was observed under a light microscope at low power (10x).

-Statistical Analysis 

The data analysis was conducted using IBM SPSS Statistics for Windows version 19.0 (IBM Corp., Armonk, NY, USA). The fungal growth results were presented as means and standard deviations, and the differences in the live *C. albicans* counts of the different neem concentrations were compared. An analysis of variance and post hoc Tukey’s honestly significant difference test were performed to compare the differences in the means of the observations at the various intervals and the baseline, and a *p* value ≤ 0.05 was considered to be statistically significant. The software calculated the bias representing the average discrepancy between the methods, and the upper and lower limits of agreement (LoAs) were calculated as the bias ± 1.96 times the standard deviation of the bias. The closer the bias was to zero and the narrower the LoA, the more agreement there was between the methods.

## Results

**Figure 1:**
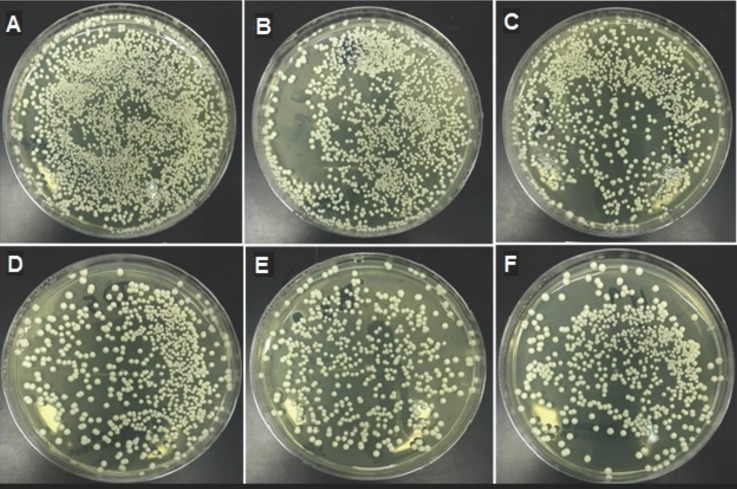
Cultures of *Candida albicans* colonies based on different neem concentrations Heat polymerized acrylic resin. (A) Control; (B) 0.5% neem; (C) 1% neem; (D) 1.5% neem; (E) 2% neem; (F) 2.5% neem.

The means, standard deviations and p values of the heat-polymerized specimens for both tests are presented in  Table 3. The addition of neem to the heat polymerized acrylic resin showed strong antifungal activity with all of the concentrations tested. Moreover, the *C. albicans* count decreased significantly as the neem concentration increased (*p*<0.000). All of the pairwise comparisons showed significant differences between the means, with the exception of the HD and HE with the direct culture method. The HE showed maximum inhibition against the *C. albicans* with values of 340.8±11.5 and 1,673.5±110.9 for direct culture and slide counting methods, respectively, when compared to the control group (2,061.4±31.1 and 9,730.5±451, respectively). Figure 1 shows a representative image of the colony formation in the direct culture method in relation to the neem concentrations. Figure 1A shows the highest colony count, which decreased as the neem concentration increased up to 2.5% (Fig. 1E).

**Table 3:**
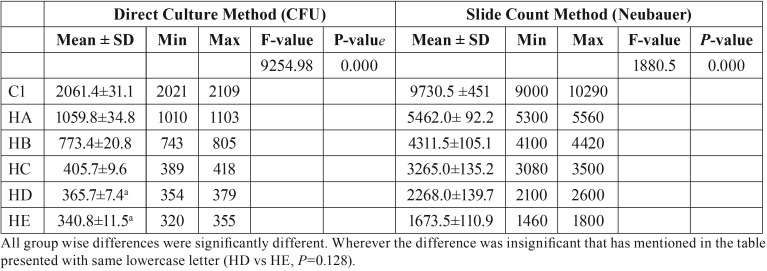
Mean, standard deviation, and *P*-values for heat polymerized specimens modified with different concentrations of neem powder.

**Figure 2:**
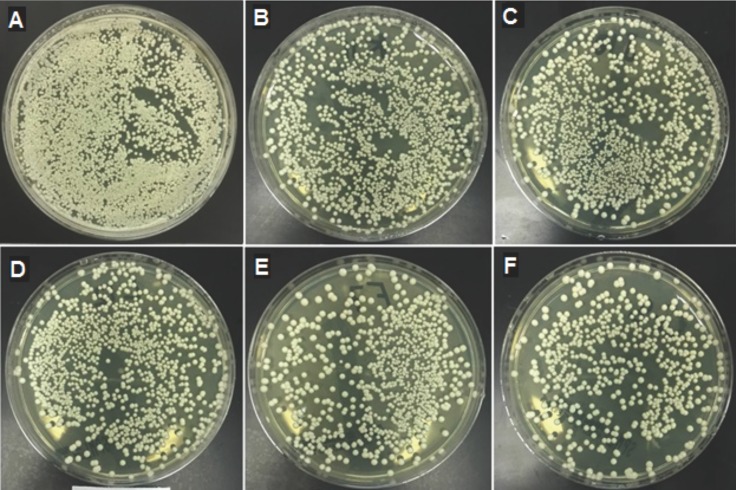
Cultures of *Candida albicans* colonies based on different neem concentrations auto-polymerized acrylic resin. (A) Control; (B) 0.5% neem; (C) 1% neem; (D) 1.5% neem; (E) 2% neem; (F) 2.5% neem.

The means, standard deviations and p values of the auto-polymerized specimens for both tests are summarized in  Table 4. The addition of neem to the auto-polymerized specimens decreased the *C. albicans* count as the neem concentration increased in all of the modified groups in both the direct culture and slide counting methods. Figure 2 shows a representative image of the colony formation for the direct culture method in relation to the neem concentrations. Figure 2A shows the highest colony count, which decreased as the neem concentration increased up to 2.5% (Fig. 2E).

**Table 4:**
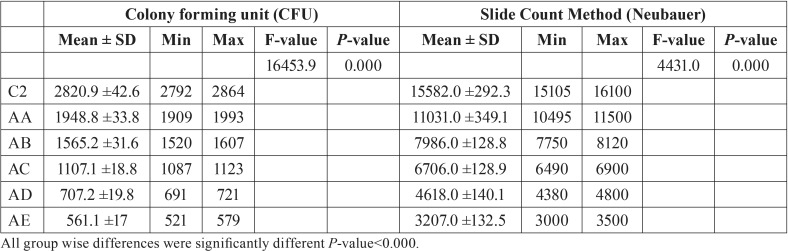
Mean, standard deviation, and *P* values for Auto-polymerized specimens modified with different concentrations of neem powder.

**Figure 3:**
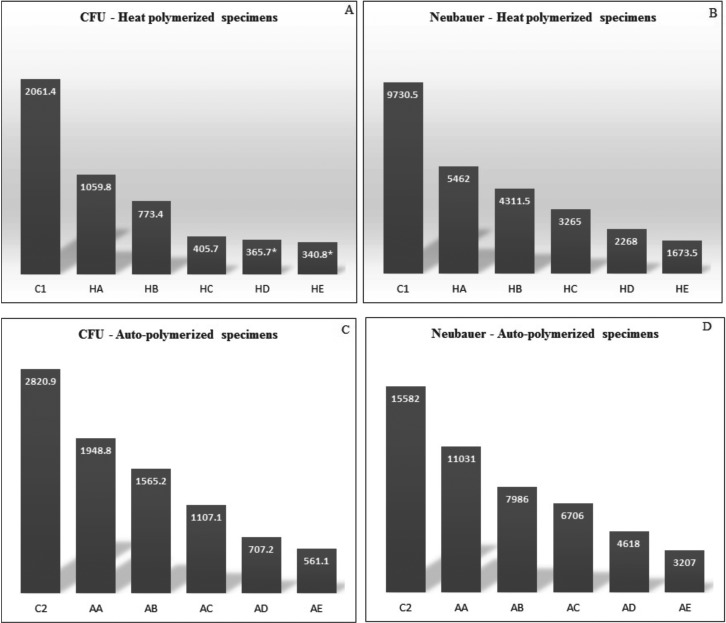
Mean values of *Candida albicans* colonies for all tested specimens according to neem concentrations.

The Bland-Altman plot in Figure 3 shows the differences in the average *C. albicans* colony count for the two methods. The bias is described as follows: as the average difference gets closer to zero, the method becomes more concordant. The upper and lower LoAs represent the bias ± 1.5 standard deviations of the bias. The upper and lower LoAs for the direct culture method and slide counting method in this experiment were -4.4 and 1.5, respectively.

## Discussion

A novel DS treatment approach was tested in the present study with the addition of neem powder to denture base materials. Its effectiveness against the adherence of *C. albicans* to the denture base materials was then tested. The results showed that the mean number of *C. albicans* colonies in the tested groups decreased significantly when compared with the conventional heat-polymerized PMMA. Therefore, the null hypothesis stating that the amount of *C. albicans* adhering to the PMMA plus neem denture materials would not be different from the amount adhering to the conventional heat-polymerized PMMA was rejected 

 Table 3 shows the slide counting method and direct culture method results, which indicated that the neem had positive antifungal effects against *C. albicans* adhesion to the heat-polymerized acrylic resin. The relationships between the neem concentrations and the *C. albicans* counts confirmed the antifungal effects of the neem. One previous study reported that because neem includes the chemical constituents of alkaloids, flavonoids, terpenoids and tannins (15), it has the ability to prevent microbial infections specifically because of its antimicrobial biological activities (21). The ability to create a complex with the bacterial cell walls can be seen in these chemicals, which demonstrates their antibacterial activity (15,21,22). Additionally, azadirachtin is a bioactive metabolite of neem that also plays a role in neem’s antibacterial potential via its inhibitory activity towards DNA topoisomerase enzyme II (22-24).

Because of the antimicrobial effects of neem powder, it has been used as an active ingredient in many toothpastes and tooth powders. Additionally, it is quite beneficial in dentistry for naturally treating gingival problems and maintaining oral health. Neem twigs have been used to relieve toothaches, as an oral deodorant, for cleaning teeth (35) and as an endodontic treatment (36). Recently, the use of neem as an endodontic irrigant was proven to be helpful due to its excellent antioxidant effects, in addition to the fact that it is highly biocompatible, with no risk of tissue toxicity during use. It has been proven that neem is biocompatible with the human periodontal ligament fibroblasts, and this is an essential factor contributing to its clinical application in endodontics (37).

In agreement with the previous study, Polaquini *et al.* evaluated the antifungal effects of a neem extract on dental composite resin, and they reported that the neem leaves showed potential antifungal effects on biofilm formation, as well as *C. albicans* adhesion and colonization (22). For all of the concentrations used in this study, the antifungal activity increased with the increase in the amount of neem. This means that the inhibition was greater on those plates with the higher neem concentrations. These findings are consistent with those of Nawasrah *et al.* (11,12) and Al-Thobity *et al.* (13), who found that the extracts of certain plants (henna and TQ) inhibited *C. albicans* adhesion at various concentrations.

An interim removable prosthesis is a prosthesis designed to improve stabilization, esthetics and/or function for a limited duration of time, and it can be substituted later with a final prosthesis (2). Auto-polymerized acrylic resin has been used in the fabrication of interim removable prostheses and orthodontic appliances, in the repair of broken denture bases (26-28), and for the fabrication of implant-supported fixed interim prostheses and maxillofacial prostheses (29). Interim prostheses provide an immediate esthetic solution until the periodontium at the extraction site is healed (30). Although auto-polymerized acrylic resin has a wide field of applications in interim prostheses, it can increase the susceptibility to *C. albicans* adhesion because of its surface properties. The importance of studying the antifungal effects of neem powder in auto-polymerized acrylic resin material is based on its versatility in clinical applications that require the use of this resin.

When comparing the values of both resins, the auto-polymerized resin showed higher values in the respective neem concentrations when compared to the heat-polymerized resin. However, the results of the two tests were the same, even with the high values reported in the auto-polymerized samples that had greater colony numbers. This is consistent with several previous studies (26,33,34), which reported that the initial microbial adhesion was considerably affected by the resin voids and roughness of the fitting surfaces of the acrylic denture base materials. This may be attributed to the fact that the auto-polymerized acrylic resin is more susceptible to *C. albicans* adhesion due to its weak surface properties, especially its excessive surface roughness when compared to the heat-polymerized resin (26). These disadvantages of cold polymerization may be clarified by the chemical initiator, which decreases the conversion degree during the polymerization stage and disturbs the surface properties (31,32).

**Figure 4:**
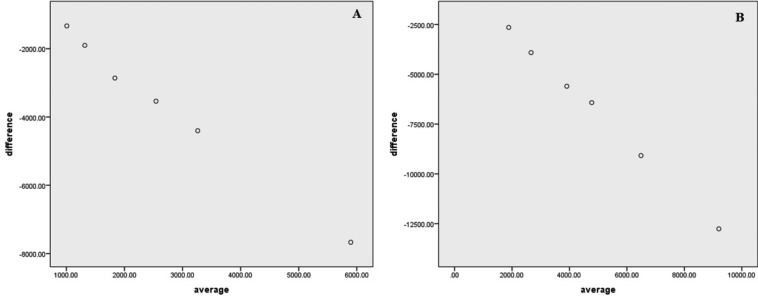
Bland–Altman Plot of colony count by two methods. A Heat, B auto.

The strengths of this study include the use of two separate antimicrobial assays and two different materials for the assessment and quantification of the *C. albicans* adhesion to the heat-polymerized and auto-polymerized PMMA plus neem denture base resins. In this study, similar log reductions with robustness and agreement were observed between the direct culture method and the slide counting method in the Bland-Altman plots (Fig. 4).

Neem is cheap, widely available, and easy to handle and apply, in addition to its antifungal activities, which indicates that the use of neem powder can help control *C. albicans* adhesion; thus, it can be used as a possible DS treatment and prevention method. Neem’s ability to inhibit *C. albicans*, as determined by this current study, should launch additional investigations determining the efficacy of neem in combating *C. albicans*. Moreover, this study may encourage the use of neem as an effective alternative method for the prevention and/or treatment of this pathogen in patients.

This study did have some limitations. For example, the oral environment contains many types of microorganisms, not just *C. albicans*. Additionally, no aging process was implemented. In future investigations with well-simulated conditions, a wide range of microorganisms, including those forming biofilms, should be assessed. Moreover, comparisons of the effects of neem and different antifungal agents on *C. albicans* adhesion, as well as investigations of the effects of neem on the physical properties of the acrylic resin denture base materials are needed.

## Conclusions

Within the limitations of this study, it can be concluded that neem powder exhibits antifungal activity by decreasing the adhesion of *C. albicans* to denture base materials. PMMA acrylic resin modified with neem could be used for the fabrication of removable prostheses as a possible DS treatment or prevention method. However, further investigations of the physical and mechanical results of adding neem to denture base materials are required.
